# Primary functioning hepatic paraganglioma mimicking hepatocellular carcinoma: A case report and literature review

**DOI:** 10.1097/MD.0000000000010293

**Published:** 2018-04-27

**Authors:** Wei Liao, Ze-yang Ding, Binhao Zhang, Lin Chen, Gan-xun Li, Jing-jing Wu, Bixiang Zhang, Xiao-ping Chen, Peng Zhu

**Affiliations:** Hepatic Surgery Center, Tongji Hospital, Tongji Medical College, Huazhong University of Science and Technology, Wuhan, China.

**Keywords:** catecholamine, hepatic paraganglioma, hepatocellular carcinoma, hyperglycemia

## Abstract

**Introduction:**

Hepatic paraganglioma (HPGL) originates from the sympathetic nervous tissue in the liver, and is an extremely rare type of the sympathetic paragangliomas. Till now, only 11 HPGL cases have been reported.

**Case presentation:**

A 49-year-old woman presented to our hospital with a lesion in the right lobe of the liver, which grew from 2 to 6 cm in 2 years. In addition, she had a 6-year history of diabetes. The patient was initially diagnosed as hepatocellular carcinoma and hepatectomy was performed. Surgical resection of the liver lesion was successful, but the blood pressure rose and fell sharply when the lesion was being removed. The pathological examination of the liver lesion showed that it was HPGL. After the operation, the patient recovered uneventfully. Follow-up examination showed the blood glucose level went back to the normal range in 20 days after the operation, and MRI and ^131^I-MIBG scan showed that there was no evidence of recurrence and metastasis in >2 years.

**Conclusion:**

By means of reporting this case and reviewing 11 reported cases, we conclude that the incidence of HPGLs is extremely low and the clinical and radiological characteristics of HPGLs are nonspecific; thus, it is hard to diagnose HPGLs correctly. Surgical resection is curative therapy for HPGLs, whereas the removing of HPGLs may cause the releasing of catecholamine, and then lead to hypertension crisis and arrhythmia. Thus, antihypertensive therapy is necessary during the operation. Follow-ups after the operation are important for HPGL patients, for pathological examinations are not sufficient to differ malignant HPGLs from benign ones, and follow-ups are helpful for HPGL patients to find the recurrent foci or metastases timely.

## 1 Introduction

Paraganglioma (PGL) is defined as a rare neuroendocrine neoplasms, and forms the main branch of pheochromocytomas and paragangliomas (PPGLs).^[1]^ PPGLs originate from the paraganglia cell clusters of neural crests and can be divided into 2 groups. Among them, the first group is defined as head and neck paragangliomas (HNPGLs) that have a close relationship with the parasympathetic nervous system. However, HNPGLs cannot produce catecholamine. They function as chemical sensors. The other group is related to the sympathetic nervous system and their location is close to sympathetic nerves that can produce catecholamine.^[2]^ According to the World Health Organization (WHO), tumors which are related to sympathetic tissues in the adrenal gland should be named as pheochromocytomas (PCCs), whereas tumors related to extraadrenal sympathetic tissues should be named as sympathetic paragangliomas (sPGLs).^[3]^ sPGL often occurs along the axis of the body, from skull-base to the pelvic floor and corresponds to the distribution of the sympathetic nerve.^[4]^ In summary, PGLs include sPGLs and HNPGLs. Research has demonstrated that in PGLs, 55.2% occurred in the retroperitoneal space, 25.6% in the head and neck, 5.6% in the bladder, and 3.2% in the mediastinum.^[5]^ However, hepatic PGLs (HPGLs) are extremely rare. To date, there has only been 11 reported HPGL cases. Among them, 7 cases were reported in China and 4 in other countries.^[6–15]^ Some HPGL patients were admitted to hospital for symptoms relating to catecholamine excess, including chronic headache, palpitation, hypertension, and so on, but others for lesions in the liver during check-up. However, it remains difficult to diagnose HPGLs correctly because their manifestation and the results of radiological examinations show no specificity as to their cause.

Herein, we report the case of a woman with HPGL who was first diagnosed with hepatocellular carcinoma (HCC). After this first diagnosis, the patient received a hepatectomy. During the operation, her blood pressure underwent severe fluctuation, especially when her tumor was removed. A final pathological examination confirmed that this tumor was PGL, and a ^131^I-MIBG scan of her entire body after surgery revealed no positive findings to support a diagnosis of HPGL.

## 2 Methods

Colored 3D reconstruction of CT images was carried out by using an IQQA-Liver reconstruction system (EDDA Technology, Shanghai, China). This study was approved by the Ethics Committee of Tongji Hospital, Huazhong University of Science and Technology. Because our case report does not violate the patient's privacy, informed consent is not necessary.

## 3 Case report

**Figure 1:**
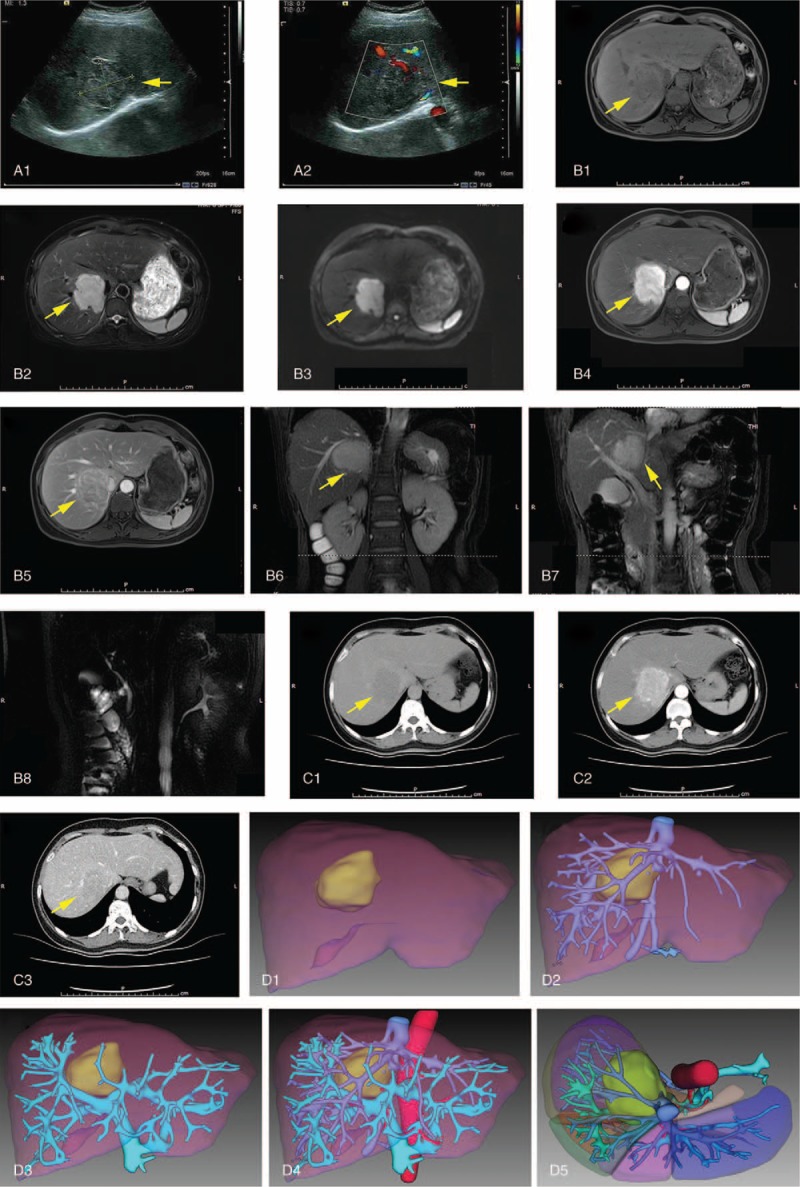
Preoperative radiological examination of the reported case. (A) Ultrasonography revealed a hypoechoic mass with its diameter of 5.7 × 4.9 cm (A1), color Doppler flow imaging indicated that the mass was next to the second porta of liver (A2). From (A) to (C), yellow arrows heads direct the liver lesion; (B) abdominal MRI scanning. T1-weighted magnetic resonance (MR) image showed a low signal intensity mass which located in the segment 7 and 8 and caudate process of the liver (B1), and T2-weighted MR image showed the lesion is of high signal intensity (B2); MR with diffusion-weighted images showed that the mass was hyperintensity with restricted diffusion (B3); MR with perfusion-weighted imaging showed the mass was hyperenhanced in the arterial phase (B4) and de-enhanced in the portal phase (B5); coronal view of 2D-FIESTA sequence images showed the mass is close to the right hepatic vein (B6) and the right posterior branch of the portal vein (B7); and MR cholangiopancreatography showed no obvious abnormality in the bile duct (B8); (C) abdominal computed tomography (CT) scanning. Plain CT scan showed a hypoattenuating liver lesion (C1); arterial phase showed nodular enhancement of the lesion (C2); portal phase showed a low density nodule(C3); (D) 3D reconstruction of CT images. anterior view (D1-D4) and upper view (D5) of the reconstructed images of liver are showed. The liver lesion is labeled with light green, liver is labeled with auburn, abdominal aorta, and hepatic artery are labeled with red, portal vein and its branches are labeled with cyan, and hepatic vein and inferior vena cava are labeled with blue.

A 49-year-old female patient presented to our hospital with a solitary lesion in her liver, which was found accidentally by an abdominal ultrasonic scan during a physical examination. This patient was asymptomatic, and physical examination of this patient showed no positive signs. The patient had no history of viral hepatitis and alcohol abuse. She had a 6-year history of diabetes and her blood glucose level was not controlled well. The blood pressure of the patient was normal. The patient found this lesion with its diameter of 2 cm in her checkup 2 years before, and she refused further examination and chose follow-up at that time. The patient had undergone breast operation 20 years before and the pathological result of the resected breast lesion was benign. The results of laboratory tests were almost in the normal range except blood glucose and α-fetoprotein (AFP). Blood glucose was significantly high (13.18 mmol/L) and AFP was a slight elevation (7.32 ng/mL, normal range: 0.605–7.0 ng/mL). The Child-Pugh score was <5. An ultrasonic scan of her liver showed the mass of the lesion to be located in the right lobe of her liver, with a size of 5.7 × 4.9 cm. The shape of the lesion was anomalistic, but its edges could be easily discerned (Fig. 1A). Magnetic resonance (MR) images showed a mass was located in the segment 7 and 8 and caudate process of the liver. T1-weighted MR images revealed that the mass is low signal intensity, and T2-weighted MR images showed the signal intensity of the mass is high (Fig. 1B1 and 2). MR with diffusion-weighted images (MR-DWI) showed that the mass was hyperintensity with restricted diffusion (Fig. 1B3), and MR with perfusion-weighted imaging (MR-PWI) showed the mass was hyper-enhanced in the arterial phase (Fig. 1B4) and de-enhanced in the portal phase (Fig. 1B5). Coronal view of 2-dimensional fast Imaging Employing STeady-state Acquisition (2D-FIESTA) sequence images showed the mass is close to the right hepatic vein (Fig. 1B6) and the right posterior branch of the portal vein (Fig. 1B7), and MR cholangiopancreatography showed no obvious abnormality in the bile duct (Fig. 1B8). CT images showed the liver mass was hypoattenuating (Fig. 1D1), and was hyperenhanced in the hepatic arterial phase (Fig. 1D2) and de-enhanced on delayed phase (Fig. 1D3). The mass was lobulated, but its edges were also discernible. The presence of calcification and expanded bile ducts were not found. On further inspection, the inferior vena cava was observed to exert a pressing force on the mass. We reconstructed 3D images from CT images to show the relationship between the mass and the portal vein, hepatic vein, and their branches (Fig. 1E). These characteristic results suggested that the mass in the right lobe of the liver was most likely HCC.

**Figure 2:**
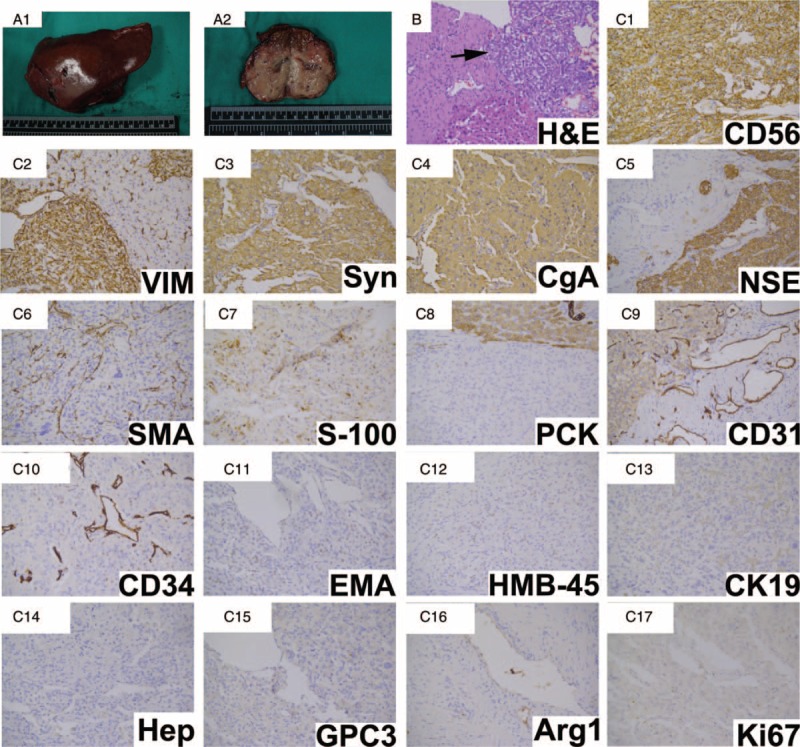
Postoperative pathological examination of the reported case. (A) Gross appearance showed the cut surface of the mass; (B) Hematoxylin-eosin staining of tumor tissue (black arrow). Original magnification: 200×; (C) immunohistochemical staining for indicated markers. Original magnification: 200×.

With sufficient preoperative preparation, the patient underwent surgical resection to remove the mass. During exploration of the abdomen, we discovered the mass to be located at segment 7 and 8, with a diameter of about 6.0 cm. The mass was observed to cling onto the second porta of the liver. Other abnormalities in the abdomen were not observed. Next, we separated the right lobe of the liver and discovered that the blood pressure of the patient suddenly increased to 220/112 mmHg. We immediately paused the operation and the blood pressure decreased to 108/60 mmHg. We noticed that the levels of blood pressure were increased to very high when the tumor was stimulated. We then exposed the retroperitoneal area of the patient and ensured no positive findings in her adrenals were present. Accordingly, we speculated that the tumor had the same characteristic of adrenal PCCs. We used large doses of sodium nitroprusside to control the patient's blood pressure and resected the tumor quickly. The blood pressure suddenly decreased to 35/20 mmHg when the tumor was totally removed, and large doses of noradrenaline were then administrated. Finally, the patient's blood pressure increased to 90/60 mmHg without pressor agents. Histopathological examination of the resected tumor discovered that it had a gray appearance with a diameter of about 6 cm (Fig. 2A). Hematoxylin-eosin (H&E) staining of the tumor tissues showed that tumor cells were irregular and with pink cytoplasm and abundant granular, sustentacular cells present, with the vascular net surrounding tumor cell nests (Fig. 2B). Immunohistochemical staining showed that the tumor tissue was positive for neural-related markers such as CD56 (neural cell adhesion molecule), synaptophysin (Syn), chromograninA (CgA), neuron-specific enolase (NSE), S-100, and tissue were also positive for several epithelial or mesenchymal markers including pan-cytokeratin, smooth muscle actin (SMA), and vimentin, whereas the tissues were negative for markers of hepatocytes such as Hepatocyte, Glypican-3, and Arginase-1, and were negative for HMB-45, a marker of melanocytic tumors. The tumor tissue was also negative for some epithelial markers such as ethidium monoacide (EMA), and cytokeratin 19. CD34, and CD31 were stained positive in vessels of tumor tissues, and Ki-67 index was in the low value (<1%) (Fig. 2C). Collectively, these results indicated that the pathological diagnosis of the tumor was PGL.

Eight days following the operation, the patient recovered without complications. In addition, blood glucose levels of this patient were decreased to the normal range within 20 days afterwards. Follow-up examination of liver function, blood glucose levels, and an MRI scan of the entire abdomen and ^131^I-MIBG scan of the patient's body showed no abnormalities. To date, the patient has followed-up for >2 years and no evidence of recurrence or metastasis has been noted.

## 4 Discussion

Although the liver is the second most frequent site of metastasis caused by malignant PCC, the incidence of HPGL is extremely uncommon. We reviewed HPGL cases in the literature of PubMed, Wanfang database, and China National Knowledge Infrastructure database, and the results are presented in Table 1. Among the 11 reported cases of HPGL, 3 patients reported no uncomfortable feelings, 3 patients reported headaches, and 4 patients showed abdomen and endocrine symptoms. Most patients were aged between 40 and 50 years’ old, and their AFP levels were in the normal range. Most of the reported cases (7/11) were first misdiagnosed as hepatocellular carcinoma (HCC). These results, as well as the diagnostic processes of this case, suggested that it was difficult to distinguish HPGLs from HCCs because the manifestations and the radiologic features of HCCs and HPGLs were similar. Although HPGLs are difficult to distinguish from HCCs, there still exists several differences to assist doctors in distinguishing between them: about 90% of HCC patients have a history of HBV or HCV infection, but this was not common to HPGLs; serum AFP levels were elevated in about 70% of HCC patients, but was normal in HPGL patients; HPGL patients showed manifestations of catecholamine excess, including hypertension, headache, palpitations, diaphoresis, and hyperglycemia. By contrast, HCC patients manifested hypertension, diabetes, hypertension, or hyperglycemia. However, these symptoms were not related with HCCs per se. As the differentiation of HPGLs and HCCs remains difficult, liver biopsy and pathological examinations are an accurate and useful method for diagnosing. In the reported 11 HPGL cases, only 2 cases received correct diagnosis before surgery by needle biopsy. However, the necessity and safety of the biopsy before surgery requires further investigation because surgical resection is the first choice for both HPGLs and HCCs, and liver biopsy in HPGL patients may lead to rupture, bleeding, or hypertension crisis.

**Table 1:**
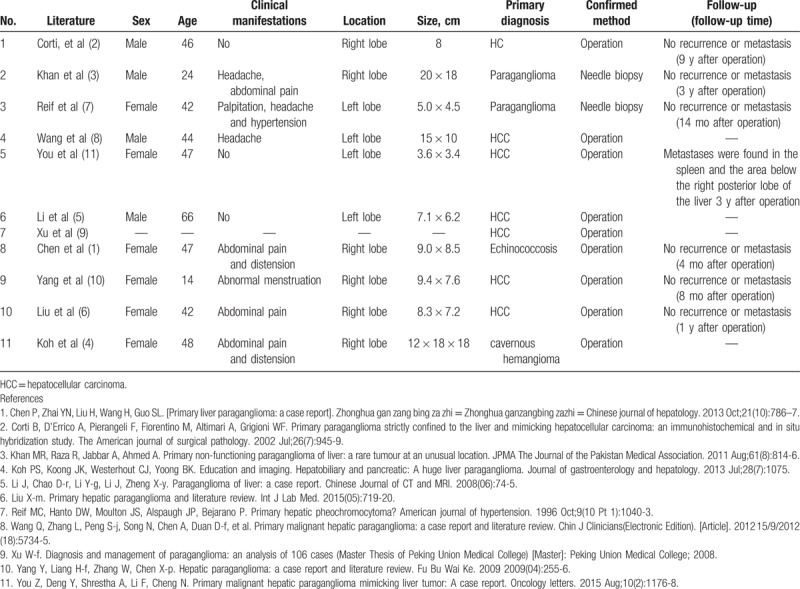
Literature review of primary paraganglioma of the liver.

Symptoms of catecholamine excess are a characteristic manifestation of sPGLs, although approximately 10% to 15% of sPGLs are asymptomatic.^[3]^ In this case, the patient suffered from hyperglycemia and was diagnosed as having diabetes for 6 years before surgery, and levels of blood glucose were gradually turned into normal after the operation, which suggested that hyperglycemia is induced by catecholamine release. In the reported 11 HPGL cases, 2 showed headache, 1 showed both headache and palpitation, and 1 female patient showed abnormal menstruation. These symptoms may be associated with catecholamine hypersecretion. Other cases (7/11) showed no significant symptom or uncharacteristic symptoms, including abdominal pain and distension. Additionally, several dormant symptoms of catecholamine excess may reveal themselves when HPGL is irritated. For example, when HPGLs are surgically removed, patients always endure a severe fluctuation of blood pressure caused by the hypersecretion of catecholamine. This phenomenon occurs even in HPGL patients presenting with no symptoms of hypertension before surgery. In this case, although the patient had no history of hypertension, we observed the drastic increase and decrease of blood pressure during surgical resection of liver mass. Similar phenomenon was also observed and reported by You et al.^[7]^ Therefore, it is necessary for doctors to prepare antihypertensive agents (α-blocker and β-blockers) to prevent or mitigate complications, including hypertensive crisis and arrhythmia during surgery.^[3]^ Finally, it is important for surgeons to remove the lesion carefully to reduce irritation to HPGLs.

The characteristic of CT imaging of HPGL is similar to that of HCC, which showed hypoattenuating lesions in the liver, and these lesions were enhanced in the hepatic arterial phase of the CT scan, and de-enhanced in the portal venous and delayed phase. In huge HPGLs, CT scanning of the liver showed that the area of contrasted enhancement lies mainly in the periphery of the liver lesion, which was consistent with hemangioma of liver.^[16]^ These characteristics have a close connection to the abundant vascular networks in tumors. The characteristics of enhanced MRI scanning for HPGLs is similar to that of CT scans; thus, MRI scans have difficulty in distinguishing HPGLs from HCCs. The most valuable examination for PGL is^ 131^I-metaiodobenzylguanidine (^131^I-MIBG) scintigraphy. It has been previously demonstrated to show a high specificity and sensitivity for detecting PCCs, and its sensitivity is 77% to 95% and specificity is 95% to 100% to PGLs.^[17]^ In addition, somatostatin receptor imaging is often used for diagnosing PGL. PGLs belong to NETs, and somatostatin receptors are expressed on the surface of tumor cells. Although octreotide signed by radionuclides can find tumors, several neuroendocrine tumors have somatostatin receptors; thus, the sensitivity and specificity of this method are lower than ^131^I-MIBG scintigraphy.^[18]^ Although PET-CT has obvious advantages in malignant tumors, it shows no specificity in PGLs. Previous studies reported that the sensitivity of ^18^F-levodopa to malignant PGL reaches 100%,^[18]^ but this result requires confirmation by further studies.

The diagnosis of HPGL depends on pathological examination. According to the literature, most PGLs showed a round but not lobulated appearance, and were often solid or hydatidiform in shape. The pathological characteristic of HPGL involves chief cells forming nests and sustentacular cells surrounding them. In addition, there can be abundant sinus-like vascular networks among these nests. Immunochemical staining revealed that CgA and NSE exert positive effects for chief cells, with an accuracy of 100% if they are both used. The expression of S-100 is positive in supporting cells.^[19]^ Furthermore, we can exclude the possibility of renal cell carcinoma if CgA, CD56, and Syn are positive, and CK and EMA are negative. HCC will be eliminated if AFP, hepatocyte, and EMA are negative. Perivascular epithelioid cell tumor (PEComa) is not supported if HMB45, CD34, and SMA are negative.

Previous investigations reported that 10% to 20% of PCCs and PGLs are malignant.^[4]^ Disappointingly, even pathological examination is not sufficient to distinguish benign from malignant PCC/PGLs, which makes the diagnosis of malignant PCC/PGLs difficult because they are routinely based on the presence of distant metastases during follow-up.^[20]^ We summarized the follow-up data of 11 reported HPGL cases 3 years after operation and discovered that only 1 case involved metastases in the spleen and the area below the right posterior lobe of the liver. Interestingly, this patient was diagnosed as having a malignant HPGL (Table 1).^[7]^ However, data or reported experience in treating metastases of malignant HPGLs were not recorded, perhaps because the incidence of malignant HPGLs is significantly small. Current therapies for malignant PCC/PGLs, including surgical resection, external-beam radiation therapy, MIBG therapy, chemotherapy, and molecular targeted therapies, may also show potential therapeutic effects on malignant HPGLs, whereas these effects need to be confirmed by further investigations.

## 5 Conclusion

The present case study, as well as those reported in the literature, revealed that HPGLs are extreme rare tumors originating from sympathetic nervous tissues in the liver, and the diagnosis and treatment of HPGLs remain challenging: some HPGL patients displayed manifestations of catecholamine excesses, whereas others showed no symptoms or nonspecific symptoms like abdominal distension. Characteristic CT and MRI imaging of HPGL were similar to that of HCC or hemangioma present in the liver. These results present challenges to the diagnosis of HPGLs because it is oftentimes difficult to distinguish HPGLs from other liver lesions; surgical resection is the first choice for treating HPGLs, whereas the stimulation of HPGLs by surgery can cause significant increases and decreases of blood pressure accounted for by catecholamine. The risk of fluctuations in blood pressure during the removal of HPGLs means that antihypertensive agents should be prepared before the operation, and surgeons should have sufficient patience and carefulness in removing HPGLs; it is difficult to distinguish malignant HPGLs from benign ones, even through pathological examination. The most effective method for diagnosing malignant HPGLs is follow-up after operation. If recurrent or metastatic foci are found during follow-up, HPGLs should be considered malignant. Finally, as there exists a lack of experience in treating metastases of malignant HPGLs, surgical resection, external-beam radiation therapy, MIBG therapy, chemotherapy, and molecular targeted therapies ought to be considered for treating HPGLs.

## Acknowledgments

The authors are grateful to Dr. Si-su Yuan (Department of radiology, Tongji Hospital) for her technical assistance in analyzing CT and MR images of this case. We also thank Dr. Dong Kuang (Department of pathology, Tongji Hospital) for his help in analyzing immunohistochemical results of this case. In addition, we thank CureEdit Bioscience Ltd. (Houston, TX) for the assistance in revising this manuscript.

## Author contributions

**Conceptualization:** Peng Zhu.

**Data curation:** Ze-yang Ding, Wei Liao, Lin Chen, Bixiang Zhang, Peng Zhu.

**Formal analysis:** Ze-yang Ding, Wei Liao, Lin Chen, Xiao-ping Chen, Peng Zhu.

**Funding acquisition:** Ze-yang Ding.

**Investigation:** Ze-yang Ding, Wei Liao, Binhao Zhang, Lin Chen, Jing-jing Wu, Gan-xun Li.

**Methodology:** Binhao Zhang.

**Supervision:** Bixiang Zhang, Xiao-ping Chen, Peng Zhu.

**Writing – original draft:** Ze-yang Ding, Wei Liao.

**Writing – review & editing:** Bixiang Zhang, Xiao-ping Chen, Peng Zhu.
